# Operationalizing Surveillance of Chronic Disease Self-Management and Self-Management Support

**DOI:** 10.5888/pcd15.170475

**Published:** 2018-04-05

**Authors:** Teresa J. Brady, Jeffrey J. Sacks, Albert J. Terrillion, Erin M. Colligan

**Affiliations:** 1 Division of Population Health, Centers for Disease Control and Prevention, Atlanta, Georgia; 2 Sue Binder Consulting, Inc, Decatur, Georgia; 3 Walden University, School of Health Sciences, Minneapolis, Minnesota; 4 Center for Medicare and Medicaid Innovation, Centers for Medicare & Medicaid Services, Baltimore Maryland

## Abstract

Sixty percent of US adults have at least one chronic condition, and more than 40% have multiple conditions. Self-management (SM) by the individual, along with self-management support (SMS) by others, are nonpharmacological interventions with few side effects that are critical to optimal chronic disease control. Ruiz and colleagues laid the conceptual groundwork for surveillance of SM/SMS at 5 socio-ecological levels (individual, health system, community, policy, and media). We extend that work by proposing operationalized indicators at each socio-ecologic level and suggest that the indicators be embedded in existing surveillance systems at national, state, and local levels. Without a robust measurement system at the population level, we will not know how far we have to go or how far we have come in making SM and SMS a reality. The data can also be used to facilitate planning and service delivery strategies, monitor temporal changes, and stimulate SM/SMS–related research.

## Why Is Surveillance of Chronic Disease Self-Management and Self-Management Support Important?

Sixty percent of US adults have at least one chronic condition, and more than 40% have 2 or more chronic conditions (1). Individual self-management (SM) of chronic conditions, also called self-care, is central to effective disease management. The Chronic Care Model identifies productive interactions between patients with the knowledge, skill, and motivation to participate in their care (also known as “activated patients”) and their proactive practice team as critical to achieving positive functional and clinical outcomes (2). Self-management support (SMS), or efforts to support individuals in their SM, is one of the Chronic Care Model’s pillars of quality improvement needed to effectively manage chronic conditions in health care (2). A robust measurement system for measuring SM/SMS at the population level could be beneficial for assessing whether the nation is making progress in SM/SMS. The data could be used to facilitate planning and service delivery, monitor temporal changes, and stimulate SM/SMS–related research.

Ruiz and colleagues identified concepts that could be developed into SM/SMS surveillance indicators (3). These concepts spanned 5 socio-ecological levels (individual, health system, community, policy, and media), mirroring Frieden’s pyramid of public health impact (4) and reflecting the multiple dimensions of SMS (5). We attempt to operationalize Ruiz et al’s concepts (3) and, where they were not feasible for surveillance, we propose new indicators that can be used to assess status, trends, and disparities in SM/SMS at the local, state, and national levels.

## Moving From the Forest to the Trees — Operationalizing Surveillance of Self-Management and Self-Management Support

We adopted Ruiz et al’s definitions of SM/SMS (3). SM consists of the tasks people must do to live well with chronic conditions and to pursue the life they desire (3,6). SMS is the systematic provision of education and supportive interventions by health care providers and others to strengthen patients’ skills and confidence in managing their health problems (ie, things done by others to support individuals in their SM efforts) (6).

We sought surveillance indicators that were sensitive to change, were actionable, had easily definable numerators and denominators, had face validity, were low-cost, required minimal effort to collect and analyze data, and — most importantly — had an existing system available to collect the data. If following a recommendation from Ruiz et al (3) was not feasible per these criteria, we explored other possible indicators to identify ones with the desired characteristics.

To standardize surveillance, we adopted the definitions in the strategic framework for multiple chronic conditions (7). We debated attempting surveillance of a single chronic disease or all chronic diseases. Should we select a single condition (like diabetes) and ascertain SM/SMS for that “sentinel” as an indicator of what is happening in other conditions? Although single conditions are likely to have specific SM activities (eg, a person with diabetes testing their blood sugar), our notion of SM addresses cross-cutting dimensions rather than disease-specific activities. Thus, we concluded that we needed to measure SM/SMS dimensions relevant across all chronic diseases, because focusing on a single condition might not be representative.

For each indicator proposed by Ruiz et al, we attempted to craft a question and specify the potential data source and numerator and denominator necessary for calculation of the indicator. As we attempted to operationalize the Ruiz et al concepts (3), we encountered consistent feasibility problems. First, we had difficulty defining a “case” of SM or SMS (the numerator) and the relevant denominator. We also lacked existing data-collection systems. The remainder of the article summarizes our deliberations on the indicators we discarded (Table 1) and our recommendations for indicators at each socio-ecological level (Table 2).

**Table 1:** Surveillance Concepts and Associated Measurement Challenges in Operationalizing Surveillance of Self-Management and Self-Management Support

Socio-Ecological Level and Concept Indicator	Measurement Challenges
**Individual Level**	
Broad issues with concepts proposed	All measures involve obtaining data from individuals with chronic disease via survey. Need to determine whether focus should be on 1) intention or action; 2) optimal SM versus some SM versus no SM; 3) whether was done within a recent period or ever done; and 4) SM for all conditions, each condition, or just one condition (SM practices might vary by condition).
Proportion and characteristics of individuals who can articulate setting a health-related SM goal and related action plans (3)	Requires ascertaining whether the person has a goal and an action plan. What if a person had one but not the other? Articulating a goal is not the same as doing (intention vs action). Uncertain which individual characteristics to select and why.
Proportion of individuals attending a series of SM education sessions in a health care setting that help solve health-related problems (3)	Uncertain whether a series of sessions is necessary or whether one session is enough. Unclear why sessions are restricted to a health care setting.
Proportion of individuals who report receiving support for or assistance with their SM goals in the past year (3)	Must first establish whether the person has a goal(s). Unclear what constitutes support or assistance. Unclear whether once is enough or if it must it be ongoing. Long recall period.
Proportion of individuals who report an improvement in their chronic disease (3)	Unclear how to link the improvement to SM.
Proportion of individuals with chronic disease who feel confident they can manage their health	Unclear how managing health differs from managing a chronic condition. Do general wellness behaviors qualify? Should confidence be measured with a binary or scalar response option? If scalar, what should be the cut point?
Proportion of individuals with chronic disease who report they have solved a problem related to their health in the past year	Unclear whether solving a problem related to health is different from solving a problem related to a chronic disease. Doesn’t necessarily equate to self-managing a chronic condition. Impact of solving just one problem unclear. Long recall period.
Proportion of individuals with chronic disease who regularly self-monitor their chronic disease symptoms	Unclear how to define “regularly.” Meaning of “self-monitoring” is unclear; for hypertension it may mean taking blood pressure, for diabetes it may mean measuring blood sugar, for conditions associated with pain it may mean quantifying pain on a scale. Does some sort of record or log need to be made? Unclear whether monitoring is the same as managing.
**Health System Level**	
Broad issues with concepts proposed for this level	Confidentiality and access issues associated with accessing medical records. Ongoing changes in electronic health records and absence of standardized systems. Unclear how to define or enumerate a health care system. No existing data collection system for measuring linkage to community resources, individual interaction with clinicians, and health care workforce training and monitoring.
Proportion of systems that incorporate SM support as part of their quality improvement plan (3)	Likely proprietary data. Unclear how to define SMS in this context.
Proportion of individual practices that track patient SM goal-setting and goal attainment and progress in the medical record (3)	Enumerating all individual practices is not feasible. Unclear why group practices excluded. Unclear what to look for in a patient health record and whether it needs to be updated at each visit. Unclear what constitutes tracking — recorded at every visit, some visits? At how many?
Proportion of accredited PCMH delivering SM support at least 50% of the time (3)	High percentage of providers choose not be a part of the PCMH quality improvement process. PCMH standards were established in 2011 and revised in 2014 and 2017; SM elements have changed in each revision. The 2017 standards contain 2 core elements (Team-based Care-9, and Performance Measurement-14) and one elective element (Knowing and Managing-22) that are too inclusive to assure that they reflect SMS.
Proportion of health care systems that link to community resources offering SM support (eg, direct referral to programs, follow-up to see if an individual attended) (3)	What if resources were offered by the system and there was no need for a community link? Unclear what would qualify as a link or which community resources it should be linked to. Unclear how data would be ascertained.
Proportion of individuals who engaged in a process with a health system that significantly changed their ability to manage their health problem (3)	Implies a survey of patients in a system. Unclear what individuals would be asked in order to ascertain whether change was significant. Unclear what is considered a process.
Proportion of health care professionals who received training on working with patients to set and monitor self-management goals (3)	Unclear what type of training should be included or whether it would differ by specialty. One trained person in a practice may be enough. Unclear denominator — which health care professionals?
Proportion of PCP practices that have provision of SM support written into staff job descriptions (3)	Unclear how to enumerate PCP practices. Unclear denominator — PCPs? Practices? Unclear how job descriptions would be gathered or if such information would be in a job description. Job descriptions may not reflect actual practice. Access and confidentiality issues associated with obtaining job descriptions.
**Community Level**	
Broad issues with concepts proposed for this level	Unclear what defines a community or community-based program. The more broadly SMS programs are defined, the more difficult it is to enumerate them. Unclear how to enumerate programs in a community. Uncertain how to count Internet-based programs. If focused on a “sentinel” delivery system (eg, YMCAs, senior centers), unclear how to determine if these facilities offer SMS programs.
Proportion of SM education/SM support programs by organization types in given counties (3)	Unclear what defines an SM education program or an SM support program. Unclear which organizations should be included and how they would be found and enumerated. How would a program be defined (eg, does an ongoing exercise class count the same time as a one-time offering of an SMS class)? Unclear denominator. Implies multiple surveys of community organizations.
Proportion of communities actively promoting the construction of supportive environments that encourage people to be active (3)	Unclear how to define active promotion, construction, a supportive environment, and encouraging being active. Unclear how to define a community and if communities of different sizes counted equally. Unclear data source.
Proportion of communities that actively promote programs that offer affordable healthy foods (3)	Unclear what specifically defines active promotion and a program that supports healthy affordable food. Unclear what defines affordable or what happens if healthy food is offered but expensive. Unclear data sources.
Proportion of communities that have infrastructure/partnerships for organizations in the community to work together to foster SM among people with chronic diseases (3)	Unclear how to define “fostering,” “community,” “infrastructure,” “partnership,” or “working together” (once? ever? ongoing?). Unclear data sources.
Proportion of individuals being encouraged to attend community programs (3)	Unclear how “being encouraged to attend” and “community programs” are defined. Unclear how data could be collected reliably.
Proportion of population with chronic disease with SM support or education programs available within 5 miles of home (20 miles for rural areas)	Difficulty geo-locating people with chronic disease. People with chronic diseases may not be distributed the same as total population. Need geo-location of person and program to measure distance from person to program. Access to programs has multiple possible definitions. If access is an issue, is it immediate proximity (distance between a location and closest program), availability in an area (number of programs within an area), availability in immediate area (number of programs within a given distance of a point), or average distance between a location and all (or individual) SMS programs?
Proportion of community benefit surveys that address the availability and/or quality of SM support programs	Lack of standard data collected by hospital associations. No one place to enumerate all the surveys that have been done.
**Policy Level**	
Broad issues related to health care systems	Unclear what defines a health care system and how could these systems be enumerated. Unclear data source.
Broad issues related to health insurance	The health insurance marketplace is complex with a number of types of insurers (government, employer, and commercial) and varied policies (individual, family, group); all have different requirements and benefits packages. Most employer-related health insurance benefits packages are inaccessible for review by nonemployees, and the number of employers is not feasible to monitor. Besides DSMT, unclear whether SMS programs for other diseases are covered. CPT or HCPCS codes are used for billing purposes and could be monitored to detect changes in health care delivery. CPT and HCPCS codes other than for DSMT are generic and are used for various activities, not specific to SMS.
Broad issues related to professional practice guidelines	Many chronic conditions (eg, cancer, chronic kidney disease, dementia, arthritis) entail multiple subconditions for which guidelines may vary. Even a discrete condition may have varying treatment guidelines. Unclear whose guidelines should be used as the standard.
Proportion of health plans financing/reimbursing for SM support (3)	No easy way to get this information from plans. Unclear what constitutes SMS in this context. No specific CPT code for SMS. Unclear how to count plans for the denominator.
Proportion of health care systems/plans including pay-for-performance incentive tied to the delivery of SM support (3)	Likely proprietary information. Unclear what constitutes a health care system and how these systems are distinct from plans. Unclear how to enumerate all plans and systems.
Proportion of insurance benefit packages that include SM support benefits (3)	Unclear what constitutes an SMS support benefit. Definition of an insurance benefit package is unclear.
Proportion of public health departments supporting SM support programs	Unclear what constitutes an SMS program and how to define support. Level of analysis is unclear (state? county? city?). Data source unclear. Unknown quality and timeliness of data among state health departments with program locator functions.
Proportion of insurance plans that reduce health insurance costs for improved SM by employees	Likely proprietary data. Reduce costs to whom? Why employees only? Unclear what activities constitute improved SM and how to distinguish from general wellness. Unclear how to define plan for the denominator.
Proportion of nursing schools with SM support included in curricula	No centralized repository of curricula.
Proportion of physician specialty certifications requiring SM support training	Exploration of new certification exam content (because need for exams on recertification is not consistent) in the specialties most likely to manage patients with chronic conditions (ie, internal medicine, family medicine, and preventive medicine) showed that SM is not explicitly mentioned per se in any of these exams.
Proportion of employers who provide SM support as an employee wellness benefit	Examined Kaiser Employee Health Benefits Survey (http://kff.org/report-section/ehbs-2015-summary-of-findings/); questions not specific enough to be sure they represent SMS.
Federally funded research or programs on SM	Number of results in grants.gov (www.grants.gov/web/grants/search-grants.html) was low, highly influenced by choice of keyword, and produced different results on different days.
**Media Level**	
Broad issues with concepts proposed for this level	Unclear how to determine whether an individual was exposed and whether exposure was relevant to the chronic disease. Recall period unclear (eg, ever? last month? last week?).
Proportion of individuals exposed to media campaigns locally, regionally, or nationally that promote SM, including collaborative goal-setting (3)	Unclear whether collaborative goal-setting must be included if other aspects of SM were covered. Unclear how to determine whether an individual was exposed. Denominator unclear (adults, adults with chronic condition, adults exposed to any media campaign?).
Proportion of newspaper columns or radio/television stories on SM support (3)	Unclear what elements must be present to constitute SMS content and how those elements would be detected (ie, what specifically would we search for?) Would it be disease-specific? Unclear denominator (the sum of all TV, radio, and newspaper stories?) and uncertain how those sums would be ascertained.
Proportion of individuals exposed to public health campaigns promoting SM	Unclear what constitutes a public health campaign or how to determine whether an exposure occurred.
Proportion of individuals exposed to social media campaigns promoting SM	Unclear what constitutes a social media campaign or how to determine whether an exposure occurred.
Proportion of product commercials that articulate SM as part of their product’s use	Unclear how SM would be defined in this context. Unclear how to enumerate products and commercials (billboard, print advertisement, television/radio advertisement?)

Abbreviations: CPT, current procedural terminology; DSMT, diabetes self-management training; HCPCS, Healthcare Common Procedure Coding System; PCMH, patient-centered medical home; PCP, primary care provider; SM, self-management; SMS, self-management support.

**Table 2:** Recommendations for Surveillance of Self-Management and Self-Management Support at Each Level of the Socio-Ecological Model

Indicator, Rationale, Data Source	Operationalization
**Individual level**	
**Indicator: **Proportion of individuals with one or more chronic conditions who report taking action within the past 3 months (or alternate reference period) to improve their most concerning chronic condition. **Rationale: **Taking action to solve problems and adapt to a health condition’s fluctuations is a key and essential feature of effective self-management. A yes answer implies that the person monitors his/her condition and takes action to maintain quality of life. **Potential data source:** Surveys that assess chronic disease status such as National Health Interview Survey (https://www.cdc.gov/nchs/nhis/index.htm), Behavioral Risk Factor Surveillance System (https://www.cdc.gov/brfss/index.html), or Medicare Current Beneficiary Survey (https://www.cms.gov/Research-Statistics-Data-and-Systems/Research/MCBS/index.html). **Notes:** Responses to Q1 can also be used to calculate which conditions among those covered in a survey are most concerning to people who have multiple chronic conditions. Responses to Q2 can also be used to calculate patterns of disease trajectory (eg, among people who report arthritis as their most concerning condition, what proportion report better, worse, or stayed the same during the reference period) and show how these proportions change over time for a given condition. Q2 responses can also be combined with Q3 responses to compare proportions taking self-management action given the condition’s getting better or worse or remaining the same. Both of these secondary analyses can be used to further target self-management support interventions.	**Questions:** Only those with 1 or more chronic diseases should be asked this question. Q1. Earlier you told me that you had (fill in list of chronic diseases [eg, diabetes, arthritis, heart disease]). Which of these conditions concerns you the most? Q2. Do you feel as if your (name of condition of most concern) has gotten better, worse, or stayed the same in the last 3 months?* Better Worse Stayed the same Not sure/don’t know Refused *Three-month reference period can be reduced (eg, to one month) to match other reference periods used on the survey. Q3. Based on what you just told me, have you changed anything about how you manage your (name of condition of most concern)? Yes (optional to go to Q4) No Not sure/don’t know Refused **Numerator:** Q3 = A (yes) **Denominator: **Q2 = A + B + C + D +E (better, worse, same, don’t know/not sure, refused) **Optional 4th question if more detailed information is desired** Q4. What did you do? __________(Record verbatim). Probe: Did you do anything else? Interviewer note: type of actions could be avoiding certain foods or environmental situations, performing specific types of activities, etc.
**Secondary Indicator:** Proportion of individuals with one or more chronic conditions who report they have taken a course or class to help manage their most concerning chronic condition. **Rationale:** Provides estimate of proportion of people attending a self-management program. Can be asked in conjunction with community-level course awareness indicator question to compute proportion who are aware and attended (if they know, will they come?). Attending a class is a self-management behavior that cuts across chronic diseases; attendance is one means to the end of becoming a good self-manager. **Proposed data source**: Surveys that assess chronic disease status such as National Health Interview Survey (https://www.cdc.gov/nchs/nhis/index.htm), Behavioral Risk Factor Surveillance System (https://www.cdc.gov/brfss/index.html), or Medicare Current Beneficiary Survey (https://www.cms.gov/Research-Statistics-Data-and-Systems/Research/MCBS/index.html).	**Question:** Only those with 1 or more chronic diseases should be asked this question. Q1. Have you ever attended a course or class on how to manage your (name of condition of most concern)? Yes No (Optional to go to Q2) Not sure/don’t know Refused **Numerator:** Q1 = A (Yes) **Denominator: **Q1 = A + B + C (Yes, No, Not sure/don’t know) **Optional second question if more detailed information is desired** Q2. There are many reasons people do not participate in a course or class to help them manage their (name of condition of most concern). Why have you not attended a course or class to help you manage that condition? __________(Record verbatim). Probe: Anything else?
**Health System Level**	
**Indicator: **Proportion of chronic disease patients seen by clinician in past 7 days who have a jointly developed self-management plan. **Rationale: **Collaborating with their patients to jointly develop a self-management plan is a key self-management support strategy performed by health care providers. **Potential data source**: Survey of health care providers, such as those conducted by the Physicians Foundation (https://physiciansfoundation.org/research-insights/biennial-physician-surveys-patient-surveys/) or other state or local organizations.	**Question:** Some clinicians think it advisable for patients with chronic diseases to develop their own plan of action for managing their condition(s). Think about the patients with chronic disease you saw in the past 7 days. With what percentage of them have you or a member of your practice team ever jointly developed such a self-management plan? Fill in percentage. _______. Don’t know Refused Note to interviewer: If a range is given, record the midpoint of that range. **Numerator:** Sum of stated percentages. **Denominator: **Number of respondents who gave percentage (excludes don’t know and refused).
**Indicator: **Proportion of National Committee on Quality Assurance Patient-Centered Medical Homes (PCMH) that report including a self-management plan in individual care plans. **Rationale: **Collaborating with their patients to develop a self-management plan is a key self-management support strategy performed by health care providers; organizations that apply for PCMH recognition report this activity if they report on elective element CM8. **Potential data source: **NCQA PCMH recognition applications database (18).	**Measurement:** Organizations that report meeting NCQA’s PCMH elective element 8 (Care Management and Support Element) (CM8) (ie, includes a self-management plan in individual care plans) (18). **Numerator:** Number of organizations that report meeting elective element CM8. **Denominator: **Number of organizations that are NCQA-recognized as PCMHs.
**Indicator:** Proportion of clinicians reporting they or a member of their practice team received specialized training on how to help patients develop a self-management plan. **Rationale: **Health care providers traditionally receive little or no specialized training in SMS techniques such as motivational interviewing. **Potential data source: **Survey of health care providers, such as those conducted by the Physicians Foundation (https://physiciansfoundation.org/research-insights/biennial-physician-surveys-patient-surveys/) or other state or local organizations.	**Question:** Have you or a member of your practice team received specialized training in helping patients develop a self-management plan? Such specialized training includes motivational interviewing, stages of change, or other patient activation techniques. Yes No Don’t know Refused **Numerator:** A (yes) **Denominator:** A + B+ C (yes, no, or don’t know)
**Community Level**	
**Indicator: **Proportion of individuals with 1 or more chronic conditions who report that they are aware of courses or classes in their community to help manage their most concerning chronic condition. **Rationale: **Awareness of classes is a proxy for community availability and also the communication of such availability. If classes exist but people are unaware of them, they are effectively not available. **Proposed data source: **Surveys that assess chronic disease status such as National Health Interview Survey (https://www.cdc.gov/nchs/nhis/index.htm), Behavioral Risk Factor Surveillance System (https://www.cdc.gov/brfss/index.html), or Medicare Current Beneficiary Survey (https://www.cms.gov/Research-Statistics-Data-and-Systems/Research/MCBS/index.html).	**Questions: **Only those with 1 or more chronic diseases should be asked this question. Q1. Earlier you told me that you had (fill in chronic diseases [eg, diabetes, arthritis, heart disease]). Which of these conditions concerns you the most? Note: This question can be eliminated if individual-level self-management questions have already identified the most concerning condition. Q2. Do you know of any courses or classes in your community to help people manage (name of condition of most concern)? Yes No Not sure/don’t know Refused **Numerator**: Q2 = A (yes) **Denominator: **Q2 = A + B + C (yes, no, or don’t know)
**Secondary Indicator: **Proportion of people with a chronic condition who were aware a class existed and took it. **Rationale: **Awareness of courses or classes is necessary but not always sufficient to motivate a person to attend the course or class. This indicator can help determine if intervention efforts need to focus on raising awareness of classes (if the proportion of people who are both aware of class and took it is high) or if there is a need for further education about the value of self-management (if the proportion of people who are aware and participating is low).	**Measurement:** If individual-level secondary indicator question on class participation, and community-level question on awareness of courses are both asked, then this indicator can be computed. **Numerator:** Individual-level secondary indicator Q1 = A (yes) **Denominator: **Community-level indicator Q2 = A (yes) (was aware of classes)
**Policy Level**	
**Indicator: **Proportion of medical school curricula that include coverage of self-management/self-management support. **Rationale: **Easily searched. As SMS becomes increasingly viewed as an important tool for quality care, it will be increasingly taught in medical schools. **Proposed data source: **American Association of Medical Colleges Curriculum Inventory and Reports (https://www.aamc.org/initiatives/cir)	**Measurement: **Search medical school curricula for SMS as a specifically taught topic. **Numerator: **Number of medical schools with SMS in curricula **Denominator: **Total number of medical schools
**Indicator**: Proportion of States that include training on self-management support in Physician Continuing Medical Education requirements. **Rationale:** Easily searched; sensitive to change in state physician CME requirements. As SMS becomes increasingly viewed as an important tool for quality care, it may be increasingly required by states. **Proposed data source:** For physicians: https://www.fsmb.org/Media/Default/PDF/FSMB/Advocacy/GRPOL_CME_Overview_by_State.pdf and http://www.cmeweb.com/gstate_requirements.php For nurses: Nursing Continuing Education Requirements by State. Brookfield WI: OnCourse Learning Corporation; 2017 (http://ce.nurse.com/RStateReqmnt.aspx)	**Measurement: **Search each state’s CME requirements for mention of patient self-management. **Numerator: **Number of states requiring CME on SM **Denominator: **Total number of states
**Indicator: **Number of clinical care guidelines that include recommendations for self-management support. **Rationale:** Easily searched; sensitive to change if consistent search strategy used. As SMS becomes increasingly viewed as part of quality care, clinical guidelines should reflect the importance of doing it. **Proposed data source: **PubMed (https://www.ncbi.nlm.nih.gov/pubmed)	**Measurement: **Search strategy "disease" [all fields]) and ("self-management" [all fields] and (“guidelines” [publication type]) where “disease” is replaced with the specific chronic condition. Restrict articles to English to better reflect United States. **Numerator: **Number of guidelines including SM **Denominator:** None
**Indicator:** Number of federal rules referencing self-management. **Rationale: **Easily searched; sensitive to changes in numbers of rules about SM if consistent search strategy is used. **Proposed data source:** *Federal Register* https://www.federalregister.gov/documents/search#advanced	**Measurement: **Search *Federal Register*; restrict to Rules only. Compare results of (“self-management” and “chronic disease”) to “self-management” and “chronic condition”) to identify unique rules. Search by year. **Numerator: **Number of rules by year **Denominator:** None
**Media Level**	
**Indicator:** Number of stories in written media that mention self-management for a particular chronic condition. **Rationale: **Annual number of stories reflects media dialogue about an issue. **Notes: **Restriction to news, wire feeds, and magazines provides reasonable breadth of coverage. Can restrict to certain areas or regions and shorter time periods. **Proposed data source: **ProQuest (www.proquest.com) or similar media monitoring service.	**Measurement:** Number of stories in English containing the words “self-management” AND “condition” in a given year in newspapers, wire feeds, or magazines. Replace term “condition” with the individual chronic condition. Search by year. **Numerator:** Number of stories by year **Denominator:** None

Abbreviations: CME, continuing medical education; NCQA, National Committee for Quality Assurance; SM, self-management; SMS, self-management support.

## What Won’t and What Might Work for Surveillance of Self-Management and Self-Management Support

### Self-management at the individual level

SM at the individual level involves a variety of activities, including adopting healthy behaviors (eg, not smoking, appropriate diet, taking medications), action planning, self-monitoring, coping with emotions, managing disability, and navigating the health care system (5,6,8). SM objectives (disease control, symptom control, prevention of deterioration) vary by condition (9). In an attempt to identify core cross-cutting elements of SM, attendees at a 2014 Self-Management Alliance meeting were asked to identify observable actions that would indicate that a person was actively and effectively self-managing his or her chronic condition. (See the Alliance’s membership list in Ruiz et al [3]). The divergent written answers indicated that SM is an abstract concept defined in many ways, including many behaviors that are hard to differentiate from general wellness behavior and that are not necessarily reflective of chronic condition SM (eg, resolutions to lose weight or exercise). The most frequently reported observation reflected taking action to manage the condition. We reasoned that other mentioned items like goal-setting and action-planning are skills taught to help transform someone into a self-manager, that is, more a means to the end rather than the end itself. We felt that the essence of SM is being able to make wise decisions and recognize and respond to changing circumstances, adapting to the changes in the disease’s trend and tempo and to the complicated realities of life with chronic disease (10,11). Taking action applies to any chronic disease and encapsulates a range of activities to respond to symptoms or situations as they arise. Action reflects the embodiment and culmination of translating education, plans, and counseling in daily life (12). The general idea of taking action to improve a chronic condition or avoid making it worse offers a comprehensive, action-oriented indicator of SM.

Because many people have multiple chronic conditions, the relative importance of the conditions may shift over time (13), and because SM may differ across conditions (9), we suggest first identifying the condition that is of most concern to consistently anchor responses. We also recommend focusing on the past 3 months to lessen recall problems (Table 2). Such questions could be used in any survey that ascertains whether respondents have a chronic disease.

Several of the recommendations of Ruiz et al (3) related to self-management education, a common cross-cutting strategy to facilitate transformation into a self-manager (10). As a secondary individual-level indicator, we suggest including a question about taking a course or class (Table 2).

### Self-management support at the health system level

The most commonly recognized site for provision of SMS is within health care systems; the Institute of Medicine definition of SMS specifies only health care providers (14). SMS at this level can take the form of motivational interactions (eg, motivational interviewing), collaborative care planning strategies, tools to enhance the patient–provider interaction, self-management education programs, and referrals to community SMS resources (15). Goal-setting and action-planning feature prominently in many primary care quality improvement projects (16) and allow the clinician to help create the self-management plan (17).

Action planning, or collaborating with patients to develop an SM plan, is a key means of SMS and a key activity in most health care provider–based SMS strategies (15). Privacy concerns make it difficult to ascertain what happens in clinical encounters. Consequently, we propose an indicator to capture from health care providers how frequently action planning occurred in the past 7 days. A parallel indicator is available from the National Committee for Quality Assurance’s 2017 standards for recognition as a patient-centered medical home, which includes an elective element on developing a self-management plan (18). Because providing SMS is not a traditional part of health care provider training (19), we also recommend an indicator on specialized training in helping patients develop an SM plan (Table 2). These questions could be added to existing surveys of physicians or other health care providers.

### Self-management support at the community level

Community resources are one of the pillars of quality improvement in the original (2) and in the Expanded Chronic Care Model (20). SMS at this level could be reflected by the availability, accessibility, and awareness of SMS programs or by community structures (coalitions, parks, greenspaces, access to appropriate foods) that support SM (5,6). Clinical–community linkages could be reflected in referrals to community programs.

We encountered difficulties enumerating SMS programs and supportive community structures (Table 1). We considered identifying a sentinel SM program (eg, diabetes SM programs [21] or the Stanford Chronic Disease Self Management Program [22]) and using number of programs listed on a centralized listing of available programs (ie, a program locator) as the indicator. However, as with using SM practices for only a single disease, we were concerned with the representativeness of one program.

To examine geography-based community availability measures, we explored the use of web-based search services (eg, Google Maps) to search for nearby SMS programs. However, searches for “self-management” or “managing X condition” returned little of value. Until there is a roster of SMS programs and robust program locators, developing an indicator based on the geography of SMS programs is not feasible.

We concluded that the most feasible indicator of community-level SMS was the availability of SMS programs in the community. Directly asking people with chronic disease if they were aware of a course appeared to be the most feasible way to measure community-level SMS, because it implicitly integrates awareness and availability. This question, used in combination with a question about taking a course (see individual-level secondary indicator in Table 2), could capture both access and uptake.

### Self-management support at the policy level

Policy can be an effective facilitator of SM/SMS at all levels of the socio-ecological model and is highlighted in the Expanded Chronic Care Model (20). Many major changes in public health (eg, decreased smoking, increased use of seat belts) came from policy interventions. Policy can help reduce barriers or enhance facilitators such as SMS program availability or financing. Ruiz et al (3) suggested a focus on health care systems and health insurance policies; however, we identified multiple issues that rendered them infeasible (Table 1). Instead, we identified alternative approaches in health care professional training and certification, professional practice guidelines, and federal rules and grants.

#### Professional training and certification

Health professionals’ promotion of SM can influence patients’ use. However, most providers receive no formal training in SM/SMS (19). A content review of curriculum standards from the American Association of Colleges of Nursing (23) indicated no SMS-related standards. A survey of medical school curricula did not list SMS specifically, although some curricula included counseling for behavior change, which could be a component of SMS (24). Continuing medical education (CME) requirements for physician and nurse relicensure, on the other hand, is a potentially useful metric to follow (baseline = zero), given the ease in searching requirements and variation by state. Although some states dictate CME in certain topics (eg, pain management), none mention SMS (Table 2).

#### Professional practice guidelines

Treatment guidelines can reflect the integration of SMS into routine care delivery. PubMed provides a standardized searchable source for peer-reviewed literature, where physicians likely look for guidelines. Using the strategy shown in Table 2, we found mentions of SMS in practice guidelines for diabetes (n = 28), asthma (n = 6), arthritis (n = 4), chronic obstructive pulmonary disease (n = 1), and HIV (n = 1).

#### US government policy as reflected in the *Federal Register*

We found no ongoing inventory of federal activities or funding on SMS (Table 1). Monitoring issuance of federal government rules on SM/SMS over time can reflect policy changes. The *Federal Register* has a searchable database containing rules, notices, and proposed federal rules. Two searches (Table 2) of the database identified modest variability in numbers by year, but numbers were very low (Table 3).

**Table 3:** Number of *Federal Register* Rules Related to Chronic Condition Self-Management, by Relationship to Diabetes, 2009–2016

Year	“Self-Management” and “Chronic Disease”^a^	“Self-Management” and “Chronic Condition”^b^	Unique^c^	Diabetes-Related^d^	Not Diabetes-Related^e^
2009	2	1	2	1	1
2010	5	0	5	2	3
2011	3	1	3	1	2
2012	1	1	2	1	1
2013	1	1	2	2	0
2014	1	2	2	1	1
2015	1	0	1	0	1
2016	5	5	5	2	3
Total	19	11	22	10	12

a Number of rules found in *Federal Register* search of Rules only using search terms “self-management” and “chronic disease.”

b Number of rules found in *Federal Register* search of Rules only using search terms “self-management” and “chronic condition.”

c Number of unique rules found after manual comparison of search results “self-management” and “chronic disease” vs “self-management” and “chronic condition.”

d Number of unique rules found that were specific to diabetes.

e Number of unique rules found that were not specific to diabetes.

### Self-management support at the media level

Media coverage of SM/SMS could help set social norms and influence people with chronic disease to explore SM/SMS (5,10). We identified several impediments to exposure-based indicators proposed by Ruiz et al (3) (Table 1) but found that measuring volume of coverage was possible. Several services routinely monitor media streams (eg, newspapers, wire feeds). We searched ProQuest (ProQuest LLC) for English-language articles containing “self-management” and “condition” (specifying the list of chronic conditions [7]) from 2010–2016 in newspapers, wire feeds, or magazines. On the basis those results (Table 4), we concluded that using such a database with a consistent search strategy (Table 2) can reflect the volume of SM–related content.

**Table 4:** Number of Newspaper, Wire Service, and Magazine Stories That Mention Self-Management, by Chronic Condition, 2010–2016

Condition	Number of Self-Management Mentions^a^							
	Total	2010	2011	2012	2013	2014	2015	2016
Diabetes	4,297	502	548	685	653	728	669	512
Cancer	1,632	219	197	291	226	280	231	188
Chronic disease	1,464	166	187	221	225	252	217	196
Depression	956	124	118	151	141	160	133	129
Breast cancer^b^	933	184	89	124	150	165	130	91
Stroke	718	93	73	106	113	135	108	91
Arthritis	700	89	89	101	117	122	106	76
Asthma	400	56	44	66	56	81	60	37
Dementia	370	16	45	63	18	124	45	59
Hypertension	290	30	38	35	51	65	33	38
Autism	279	44	50	28	42	44	44	27
COPD	220	11	27	44	43	38	25	32
HIV	212	28	31	33	32	33	28	27
Osteoporosis	187	29	20	24	38	32	22	22
Chronic heart failure	105	10	10	13	25	22	18	7
Alcoholism^b^	84	6	4	16	11	26	16	6
Chronic kidney disease	75	17	1	3	9	32	9	4
Hepatitis	63	1	10	11	12	8	13	8
Autism spectrum disorder	49	3	4	5	11	9	11	6
Schizophrenia	47	7	7	6	8	5	2	12
Coronary artery disease	26	2	9	4	3	1	4	3
Hyperlipidemia	12	4	1	0	2	3	2	1
Cardiac arrhythmia	8	1	2	0	0	0	5	2
Substance abuse disorder	1	0	0	0	0	1	0	0
**Total**	13,128	1,642	1,604	2,030	1,986	2,366	1,931	1,574

Abbreviations: COPD, chronic obstructive pulmonary disease; HIV, human immunodeficiency virus.

a Numbers are from the PROQUEST database (www.proquest.com).

b Breast cancer added (to compare with cancer) and alcoholism added (to compare with substance abuse disorder) to assess using differing terms.

## Advancing Surveillance of Self-Management and Self-Management Support

We propose operationalized indicators for surveillance of SM/SMS at 5 levels of the socio-ecological model (Table 2). Measuring both SM and SMS is important, as is measuring SMS at multiple levels. SMS indicators could be considered process indicators for the eventual outcome of SM. If society effectively promotes SMS at multiple levels, more people with chronic disease may actively self-manage.

Identifying indicators that are a reasonable reflection of SM/SMS at each level was challenging (Table 1). A recurring constraint was our assumption that resources for such surveillance would be minimal. Thus, we limited ourselves to using existing surveys, systems, and databases rather than creating new surveillance systems as would have been required by the indicators proposed by Ruiz et al (3). Our proposed indicators provide only small glimpses of the big picture. Nevertheless, we believe they can provide useful information on SM/SMS status. Survey-dependent indicators will require cognitive testing.

Our proposed indicators of individual-level SM and community-level SMS rely on surveys of individuals with chronic diseases, such as the Behavioral Risk Factor Surveillance System. Local communities, health systems, or other organizations could gather data to reflect SM/SMS within their nexus of control. Surveillance of community-based SMS programs would be strengthened by development of consistent, accurate program-locator services, which could also facilitate program participation.

Two of our proposed indicators at the health system level (co-development of an SM plan and specialized training in SMS) require surveys of health care providers. Local health systems could survey their affiliated providers to assess SMS in their systems. One way to both strengthen surveillance and facilitate SMS delivery at the health system level would be the creation of Current Procedural Terminology codes within the Healthcare Common Procedure Coding System specific to chronic disease SMS (25).

We were unable to identify a strong surveillance indicator of SMS at the policy level. However, the indicators we proposed are promising because they are searchable and likely to detect change if consistent search strategies are used. Finally, we suggest surveillance of media coverage that includes mention of SM, and we identified a potential database for national-level surveillance. Similar media monitoring could be conducted at the state or local level by adding geographic restrictions to the search.

Consistent with the idea that “what gets measured gets done,” surveillance of SM/SMS can serve as both a progress report and a motivator for expansion (26). Gathering data directly from people with chronic conditions is essential to national and state surveillance to capture SM and SMS provided beyond the health care system. Inclusion of the proposed indicators into surveys like the National Health Interview Survey, Behavioral Risk Factor Surveillance System, and Medicare Current Beneficiary Survey and adding provider-based indicators to provider surveys would advance SM/SMS surveillance. Achieving the improved functional and clinical outcomes predicted by the Chronic Care Model (2,20,26) is unlikely without leadership and investment in promoting SM/SMS. Without surveillance, we will not know how far we have come or how far we have to go in making SM/SMS an integral part of health and health care.
